# Residual cholesterol is independently associated with arteriogenic erectile dysfunction: results from a multi-institutional study

**DOI:** 10.3389/fendo.2025.1715619

**Published:** 2026-01-05

**Authors:** Yangyang Mei, Yu Liu, Guoyang Zhang, Yiming Chen, Wei Xia, Mingran Zhang, Renfang Xu, Wei Zhang, Xingliang Feng, Qianfeng Zhuang

**Affiliations:** 1 Department of Urology, Jiangyin People’s Hospital, The Jiangyin Clinical College of Xuzhou Medical University, Jiangyin, Jiangsu, China; 2 Department of Urology, The Third Affiliated Hospital of Soochow University, Changzhou, Jiangsu, China; 3 Department of Urology, The First People’s Hospital of Changzhou, Changzhou, Jiangsu, China; 4 Department of Urology, The Second Affiliated Hospital of Anhui Medical University, Hefei, China

**Keywords:** erectile dysfunction, arteriogenic erectile dysfunction, residual cholesterol, lipid metabolism, risk stratification

## Abstract

**Background:**

Erectile dysfunction (ED) is increasingly recognized as an early indicator of vascular health, with arteriogenic ED (AED) being the subtype most closely linked to endothelial dysfunction and atherosclerosis. Residual cholesterol (RC), a lipid fraction carried by triglyceride-rich lipoprotein remnants, has emerged as a novel marker of residual cardiovascular and metabolic risk. However, its relationship with ED, particularly AED, has not been well characterized.

**Methods:**

From April 2023 to May 2025, men presenting with ED and controls were consecutively recruited from three hospitals. Erectile function was assessed using the International Index of Erectile Function-5 (IIEF-5), nocturnal penile tumescence and rigidity (NPTR), and color duplex Doppler ultrasonography (CDDU) to identify AED. Demographic, lifestyle, clinical, and biochemical data were collected. RC values were calculated using the formula: RC = total cholesterol (TC) – HDL-C – LDL-C. Logistic regression and ROC curve analyses were performed to evaluate the association and predictive value of lipid parameters for AED. Logistic regression analyses were performed to evaluate associations between lipid parameters and ED/AED, adjusting for potential confounders.

**Results:**

A total of 216 men with ED and 110 controls were included, among whom 118 were diagnosed with AED. RC levels were significantly higher in the ED group than in controls (0.57 ± 0.33 vs. 0.50 ± 0.18 mmol/L, P = 0.042), although the association with overall ED was attenuated in multivariable analysis (OR 1.086, 95% CI 0.997–1.182, P = 0.058). By contrast, RC remained robustly associated with AED after adjustment for age, BMI, smoking, CVD, TG, and TT (OR 1.471, 95% CI 1.275-1.697, P <0.001). In ROC analysis, RC showed the best predictive performance for AED (AUC 0.726, 95% CI 0.661–0.791), compared with TC (AUC 0.625, 95% CI 0.551–0.699) and TG (AUC 0.581, 95% CI 0.507–0.656). The optimal RC cutoff of 0.595 mmol/L yielded a sensitivity of 61.9% and specificity of 74.5%.

**Conclusions:**

RC was independently associated with AED and demonstrated stronger predictive ability than conventional lipid parameters. These findings suggest RC may serve as a useful biomarker for vascular risk stratification in men with ED, although prospective studies are warranted to validate these associations.

## Introduction

Erectile dysfunction (ED) is one of the most common male sexual disorders, characterized by the persistent inability to achieve or maintain an erection sufficient for satisfactory sexual performance (1, 2). It affects men across all age groups, with prevalence increasing significantly with advancing age, and has a profound negative impact on quality of life and psychosocial well-being (3–5). Beyond its role as a sexual health problem, ED has increasingly been recognized as a sentinel marker of systemic vascular health (6, 7). Many factors may contribute to the development of ED, among which metabolic syndrome (MS) is closely associated with ED (8, 9). MS is a multifaceted condition primarily characterized by hypertension, dyslipidemia, and impaired glucose regulation. Penile erection is primarily a vascular process, as the penis is a highly vascularized organ. Accordingly, vasculogenic ED is mainly attributed to endothelial dysfunction, which represents the most frequent form of organic ED (10, 11). Among the vasculogenic subtypes, arteriogenic ED (AED) is the one most closely associated with vascular pathology (12). It is strongly linked to endothelial dysfunction and atherosclerosis, both of which impair penile arterial inflow and compromise erection (13, 14). This vascular component positions AED within the continuum of cardiovascular disease (CVD), underscoring its importance not only for urology and andrology but also for cardiovascular risk assessment (15, 16).

Dyslipidemia is a well-established risk factor for CVD and has also been implicated in the pathogenesis of ED (17–19). Abnormal lipid profiles can impair endothelial function, reduce nitric oxide bioavailability, and accelerate atherosclerotic changes in penile arteries, thereby contributing to erectile impairment (20). Several studies have examined the association between conventional lipid parameters—such as total cholesterol (TC), low-density lipoprotein cholesterol (LDL-C), high-density lipoprotein cholesterol (HDL-C), and triglycerides (TG)—and ED (21–23). However, most of these investigations have focused on the general ED population rather than on specific ED subtypes. Dysregulation of lipid metabolism may be more relevant to AED, which is directly linked to endothelial dysfunction and atherosclerosis. Therefore, beyond HDL-C and LDL-C, the role of cholesterol levels in the occurrence of AED warrants further investigation.

In recent years, remnant cholesterol (RC) has gained increasing attention as a novel lipid-related biomarker (24). RC reflects the cholesterol content carried by triglyceride-rich lipoprotein remnants, which are mainly composed of chylomicron remnants, VLDL, and intermediate-density lipoproteins (IDL). It is typically calculated by subtracting HDL-C and LDL-C from TC (25). RC circulates in the plasma and accumulates in the subendothelial space, where it promotes endothelial dysfunction, inflammation, and ultimately the development of atherosclerosis (26, 27). Consequently, RC is now recognized as a highly atherogenic lipoprotein fraction and a surrogate marker of residual cardiovascular and metabolic risk beyond traditional lipid parameters (28, 29). Recent evidence from the NHANES cohort further demonstrated that elevated RC levels were associated with a higher prevalence of ED among men with diabetes (30). These findings highlight the potential role of RC in the pathogenesis of ED, particularly in AED, and underscore the need for further clinical investigations. In clinical and epidemiological studies, RC is most commonly estimated as total cholesterol minus HDL-C and LDL-C rather than directly measured, because direct quantification of remnant cholesterol requires ultracentrifugation or nuclear magnetic resonance spectroscopy, which are expensive, time-consuming, and not routinely available in large-scale population or hospital-based settings. The calculated RC has been validated as a reliable surrogate and strongly correlates with directly measured remnant cholesterol in previous research (31).

Despite these advances, evidence directly examining the relationship between RC and specific subtypes of ED remains scarce. In particular, the potential association between RC and AED, which is the subtype most closely linked to endothelial dysfunction and atherosclerosis, has not been systematically investigated. To address this knowledge gap, we conducted a multicenter clinical study that first compared RC levels between men with ED and controls, and then further identified AED patients using nocturnal penile tumescence and rigidity (NPTR) testing and color duplex Doppler ultrasonography (CDDU). Our aim was to evaluate whether RC is independently associated with ED, with a particular focus on its role in AED, and to explore its potential diagnostic value in distinguishing AED from controls.

## Methods

### Study population

From April 2023 to May 2025, we consecutively enrolled men (18–65 years) attending the urology/andrology outpatient clinics of three hospitals—The Third Affiliated Hospital of Soochow University, Jiangyin People’s Hospital, and The First Affiliated Hospital of Anhui Medical University—with a chief complaint of ED. Erectile function was assessed using the 5-item International Index of Erectile Function (IIEF-5) (32); a score ≤ 21 was used to diagnose ED. Eligible participants reported regular sexual activity (≥ 1 time per week) with a stable opposite-sex partner of at least 6 months’ duration, and an ED duration > 6 months.

Exclusion criteria were a history of pelvic or perineal surgery; diagnosed hormonal disorders (e.g., hypothyroidism, hyperthyroidism, androgen deficiency, or hyperprolactinemia); neurological disease; alcohol abuse; or current use of medications known to affect sexual function or lipid metabolism (e.g., antipsychotics, antidepressants, or phosphodiesterase type-5 inhibitors); Participants who were taking lipid-lowering medications (such as statins or fibrates) or androgen-modulating therapies (including testosterone replacement or antiandrogens) were excluded to avoid confounding effects on lipid metabolism and hormonal status. The control group was recruited from the health examination centers of the participating hospitals and met the same inclusion/exclusion criteria; these men reported no ED-related complaints before enrollment and had an IIEF-5 score > 21. The study protocol was approved by the local institutional ethics committees, and written informed consent was obtained from all participants prior to enrollment.

### Clinical assessment and laboratory measurements

Baseline demographic and clinical characteristics, including age, body height, weight, and body mass index (BMI), were recorded for all participants. Information on lifestyle factors such as smoking status and regular exercise (twice a week, at least 30 minutes each time), as well as medical history including CVD and diabetes, was obtained through structured interviews and review of medical records. Erectile function was assessed using the IIEF-5 questionnaire as described above.

After an overnight fast, venous blood samples were collected. Laboratory measurements included fasting blood glucose (FBG), TC, TG, HDL-C, LDL-C, VLDL, and total testosterone (TT). TC and TG were determined using enzymatic methods, HDL-C and LDL-C were measured by direct assays, and FBG was assessed using the glucose oxidase method. Serum TT concentrations were measured with chemiluminescent immunoassay (CLIA). RC was calculated as TC minus HDL-C and LDL-C.

### Erectile and vascular assessments

Nocturnal penile tumescence and rigidity (NPTR) testing was performed using the RigiScan™ device (GOTOP Inc., USA) over two consecutive nights. Participants were instructed to avoid alcohol, caffeine, and other sleep-disturbing factors and to empty their bladder before sleep. Monitoring was conducted from 22:00 to 08:00. NPTR results were considered normal if one of the following criteria was met: (i) at least three episodes of erectile events lasting ≥ 10 minutes in a single night with tip rigidity ≥ 70%; or (ii) an increase in penile circumference ≥ 3 cm at the base or ≥ 2 cm at the tip. Otherwise, NPTR was classified as abnormal (33).

**Figure 1:**
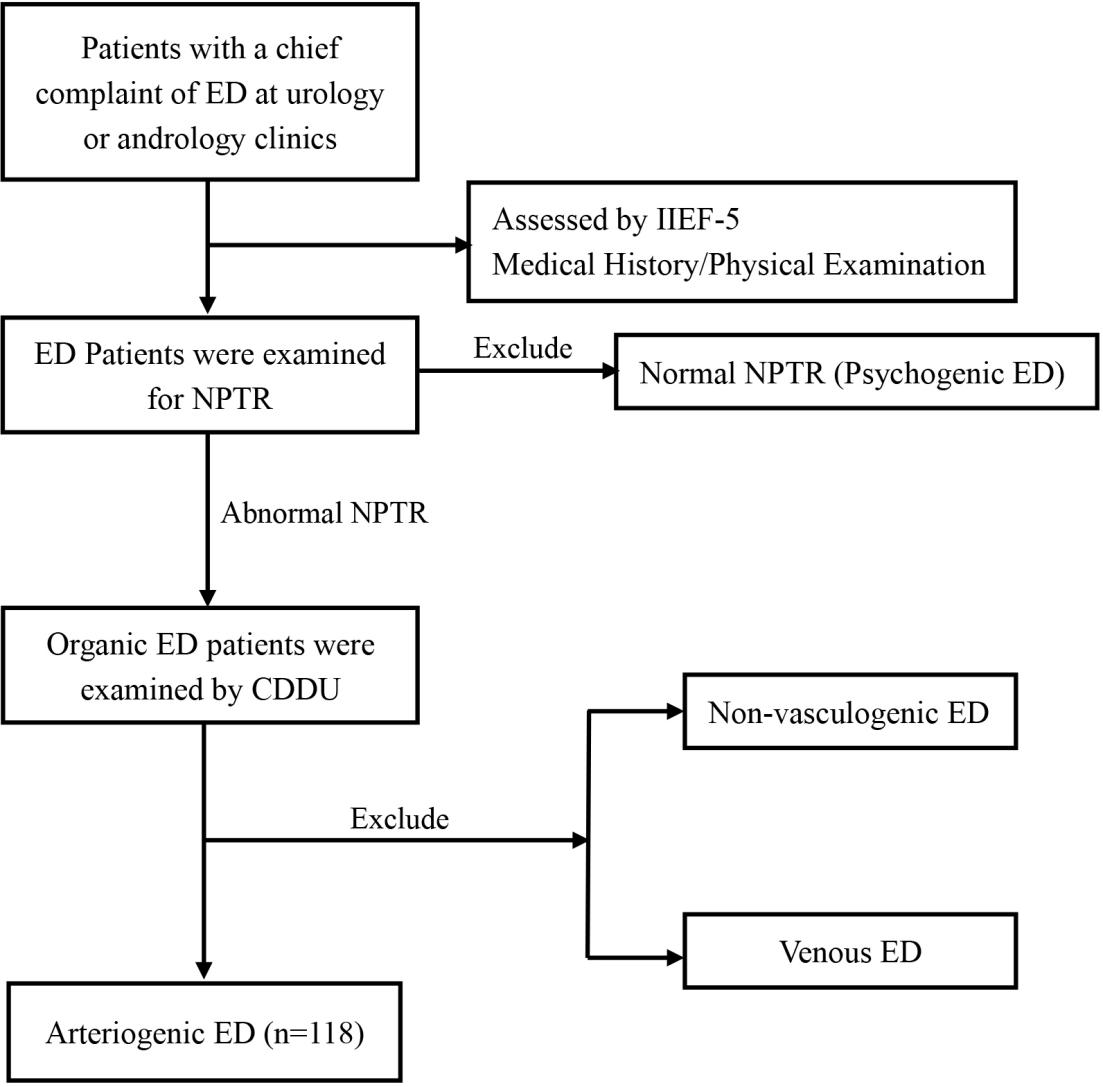
Flowchart of study population. Flow diagram showing participant enrollment, exclusion, and classification.

Men with abnormal NPTR underwent color duplex Doppler ultrasonography (CDDU). After intra-cavernosal injection of 20 μg alprostadil (Caverject^®^, Pfizer, New York, USA), penile Doppler ultrasound was performed by experienced radiologists using the Aixplorer™ system (Supersonic Imagine S.A, Aix-en-Provence, France). Peak systolic velocity (PSV) and end-diastolic velocity (EDV) were recorded at 5, 10,15, 20-, and 25-minutes post-injection. A PSV <35 cm/s was considered indicative of arterial insufficiency and used to diagnose AED. A PSV ≥ 35 cm/s combined with EDV > 5 cm/s was diagnostic of venogenic ED (VED) (34, 35). Subjects not meeting either criterion were categorized as having non-vasculogenic ED. After CDDU examination, participants were counseled regarding post-procedure precautions to ensure safety. CDDU examinations were performed at each hospital by one experienced radiologist who was blinded to participants’ clinical information. All operators received unified protocol training before study initiation, and standardized scanning parameters were used across centers. Inter-observer variation was minimized by periodic calibration and review of representative images. Intra-observer variability was assessed in 10 randomly selected participants per center, showing a coefficient of variation < 5% for both PSV and EDV. The patient selection process is illustrated in **Figure 1**.

### Statistical analysis

Statistical analyses were conducted using SPSS version 26.0 (SPSS Inc., Chicago, IL, USA) for Microsoft Windows. The Shapiro-Wilk test was applied to assess data normality. Continuous variables with normal distribution are presented as mean ± standard deviation (SD) and compared using independent-sample t tests, whereas skewed variables are expressed as median (interquartile range, IQR) and compared with the Mann–Whitney U test. Categorical variables are reported as counts and percentages and compared using the chi-square test.

Univariate logistic regression analyses were initially performed to screen potential risk factors. Variables significant at P <0.10 in univariate analyses, along with clinically relevant covariates, were entered into multivariate logistic regression models. To avoid multicollinearity, variance inflation factor (VIF) values were calculated, and only variables with VIF <2 were included in the multivariate models. To address potential confounding, we adjusted for age, BMI, smoking history, CVD, TT, and lipid parameters. For the comparison of ED patients with controls, the multivariate model included age, BMI, smoking status, CVD, TG, TT, and RC. For the analysis of AED versus controls, two separate multivariate models were constructed: Model 1 included age, BMI, smoking status, CVD, FBG, TC, TG, and TT; Model 2 included age, BMI, smoking status, CVD, FBG, TG, TT, and RC. This approach ensured that conventional cholesterol fractions and RC were not included simultaneously, thereby minimizing collinearity (all VIF <2). Odds ratios (ORs) with 95% confidence intervals (CIs) were reported.

Receiver operating characteristic (ROC) curve analysis was further used to evaluate the diagnostic performance of TG, TC, and RC for identifying AED. The area under the curve (AUC) and optimal cut-off values were determined using the Youden index. A two-sided P <0.05 was considered statistically significant.

## Results

### Baseline characteristics of participants

A total of 216 men with ED and 110 controls were included. As shown in **Table 1**, age, BMI, lifestyle factors (smoking and regular exercise), fasting blood glucose, TC, TG, HDL-C, VLDL, LDL-C, and total testosterone did not differ significantly between groups. The ED group had a markedly lower IIEF-5 score (median 12.00 vs. 23.00, P <0.001). Notably, RC was significantly higher in men with ED compared with controls (0.57 ± 0.33 vs. 0.50 ± 0.18 mmol/L, P = 0.042). The prevalence of CVD and diabetes tended to be higher in the ED group, although the differences were not statistically significant.

**Table 1:** Demographic and clinical characteristics of men with and without ED.

Variable	ED (n=216)	Control (n=110)	P
Age (years) *	33.50 (23.50-43.50)	33.00 (27.00-39.00)	0.584 **^a^**
BMI (kg/m^2^)	23.74 ± 2.65	23.52 ± 1.69	0.419 **^b^**
Personal history			
Smoking, n (%)	75 (34.72)	36 (32.73)	0.719 **^c^**
Regular exercise, n (%)	62 (28.70)	34 (30.91)	0.680 **^c^**
IIEF-5 score *	12.00 (4.25-19.75)	23.50 (22.00-25.00)	<0.001 **^a^**
Hematologic parameters			
FBG (mmol/L)	5.09 ± 0.48	5.02 ± 0.54	0.224 **^b^**
TC (mmol/L)	3.84 ± 0.62	3.72 ± 0.60	0.096 **^b^**
TG (mmol/L) *	1.32 (0.72-1.92)	1.30 (0.85-1.175)	0.897 **^a^**
HDL-C, (mmol/L)	1.16 ± 0.24	1.13 ± 0.25	0.398 **^b^**
VLDL (mmol/L) *	0.33 (0.08-0.58)	0.35 (0.14-0.56)	0.496 **^a^**
LDL-C (mmol/L) *	2.05 (1.41-2.69)	1.98 (1.45-2.51)	0.571 **^a^**
RC (mmol/L)	0.57 ± 0.33	0.50 ± 0.18	**0.042 ^b^**
TT (nmol/L)	17.26 ± 4.25	17.87 ± 4.17	0.214 **^b^**
CVD, n (%)	60 (27.78)	22 (20.00)	0.126 **^c^**
DM, n (%)	23 (15.03)	14 (12.73)	0.576 **^c^**

Values are percentages or mean ± SD unless noted otherwise. *Values are presented as median with interquartile range. Regular exercise: Twice a week, at least 30 minutes each time.

ED, erectile dysfunction; BMI, Body Mass Index; IIEF-5, International index of erectile dysfunction-5; FBG, fasting blood glucose; TC, Total Cholesterol; TG, Triglyceride; HDL-C, high-density lipoprotein cholesterol; VLDL, very low-density lipoprotein; LDL-C, Low-density lipoprotein cholesterol; RC, Residual cholesterol; TT, Total Testosterone; CVD, cardiovascular disease; DM, diabetes mellitus; ^a^ Mann-Whitney U test was used; ^b^ independent sample t tests; ^c^ chi-square test. Bold indicates P < 0.05.

### Association between lipid parameters and ED

In univariate logistic regression, RC was significantly associated with ED (OR 1.090, 95% CI 1.003–1.184, P = 0.043). Other lipid parameters, including TC, HDL-C, LDL-C, and TG, were not significantly related to ED. After adjusting for age, BMI, smoking, CVD, TG, and TT in the multivariate model, the association between RC and ED was attenuated and no longer statistically significant (OR 1.086, 95% CI 0.997–1.182, P = 0.058) (**Table 2**).

**Table 2:** Multivariate logistic regression analysis for ED (ED vs. control).

Variable	Univariate logistic regression		Multivariate logistic regression	
	OR (95%CI)	P	OR (95%CI)	P
Age	1.004 (0.975-1.034)	0.787	**/**	**/**
BMI	1.041 (0.944-1.148)	0.418	**/**	**/**
Smoking	1.049 (0.646-1.704)	0.845	**/**	**/**
CVD	1.538 (0.884-2.677)	0.127	**/**	**/**
TC	1.384 (0.944-2.031)	0.096	**/**	**/**
HDL-C	1.017 (0.583-1.899)	0.397	**/**	**/**
LDL-C	1.122 (0.690-1.825)	0.642	**/**	**/**
TG	1.154 (0.684-1.945)	0.592	**/**	**/**
TT	0.966 (0.915-1.020)	0.214	**/**	**/**
RC	1.090 (1.003-1.184)	**0.043**	1.086 (0.997-1.182)	0.058

Although several lipid parameters (TC, HDL-C, LDL-C, and RC) were included in the univariate logistic regression analyses, strong collinearity was observed among them (variance inflation factor, VIF > 2). Therefore, in the multivariate logistic regression model, the included covariates were age, BMI, smoking status, CVD, TG, TT, and RC, ensuring that no multicollinearity was present (all VIF < 2).

ED, erectile dysfunction; BMI, Body Mass Index; CVD, cardiovascular disease; TC, Total Cholesterol; HDL-C, high-density lipoprotein cholesterol; LDL-C, Low-density lipoprotein cholesterol; TG, Triglyceride; TT, Total Testosterone; RC, Residual cholesterol. Bold indicates P < 0.05.

### Baseline characteristics of men with AED and control group

As shown in **Table 3**, a total of 118 patients with AED and 110 controls were analyzed. Age, BMI, smoking status, and regular exercise were comparable between groups. Compared with controls, men with AED had significantly higher TC (3.96 ± 0.59 vs. 3.72 ± 0.60 mmol/L, P = 0.002), TG (median 1.38 vs. 1.30 mmol/L, P = 0.034), and RC (0.70 ± 0.29 vs. 0.50 ± 0.18 mmol/L, P < 0.001). HDL-C, LDL-C, and VLDL did not differ significantly. Total testosterone levels were similar between groups. The prevalence of CVD was higher in the AED group compared with controls (32.2% vs. 20.0%, P = 0.037), while the prevalence of diabetes showed no significant difference.

**Table 3:** Demographic and clinical characteristics of men with and without ED.

Variable	AED (n=118)	Control (n=110)	P
Age (years) *	34.00 (25.75-42.25)	33.00 (27.00-39.00)	0.734 **^a^**
BMI (kg/m^2^)	23.75 ± 2.34	23.52 ± 1.69	0.389 **^b^**
Personal history			
Smoking, n (%)	44 (37.29)	36 (32.73)	0.471 **^c^**
Regular exercise, n (%)	34 (28.81)	34 (30.91)	0.730 **^c^**
Hematologic parameters			
FBG (mmol/L)	5.19 (4.53-5.85)	5.02 (4.28-5.76)	0.093 **^a^**
TC (mmol/L) *	3.96 ± 0.59	3.72 ± 0.60	**0.002 ^b^**
TG (mmol/L) *	1.38 (0.89-1.87)	1.30 (0.85-1.75)	**0.034 ^a^**
HDL-C, (mmol/L)	1.12 ± 0.24	1.13 ± 0.25	0.746 **^b^**
VLDL (mmol/L)	0.36 (0.12-0.60)	0.35 (0.14-0.56)	0.347 **^a^**
LDL-C (mmol/L)	2.10 (1.45-2.75)	1.98 (1.45-2.51)	0.333 **^b^**
RC (mmol/L)	0.70 ± 0.29	0.50 ± 0.18	**<0.001 ^b^**
TT (nmol/L)	17.06 ± 4.02	17.87 ± 4.17	0.135 **^b^**
CVD, n (%)	38 (32.20)	22 (20.00)	**0.037 ^c^**
DM, n (%)	16 (13.56)	14 (12.73)	0.853 **^c^**
Penile Doppler PSV, cm/s	18.63 ± 4.96		

Values are percentages or mean ± SD unless noted otherwise. *Values are presented as median with interquartile range. Regular exercise: Twice a week, at least 30 minutes each time.

ED, erectile dysfunction; AED, arteriogenic erectile dysfunction; BMI, Body Mass Index; IIEF-5, International index of erectile dysfunction-5; FBG, fasting blood glucose; TC, Total Cholesterol; TG, Triglyceride; HDL-C, high-density lipoprotein cholesterol; VLDL, very low-density lipoprotein; LDL-C, Low-density lipoprotein cholesterol; RC, Residual cholesterol; TT, Total Testosterone; CVD, cardiovascular disease; DM, diabetes mellitus; PSV, peak systolic velocity. ^a^ Mann-Whitney U test was used; ^b^ independent sample t tests; ^c^ chi-square test. Bold indicates P < 0.05.

### Logistic regression analysis of risk factors for AED

**Figure 2:**
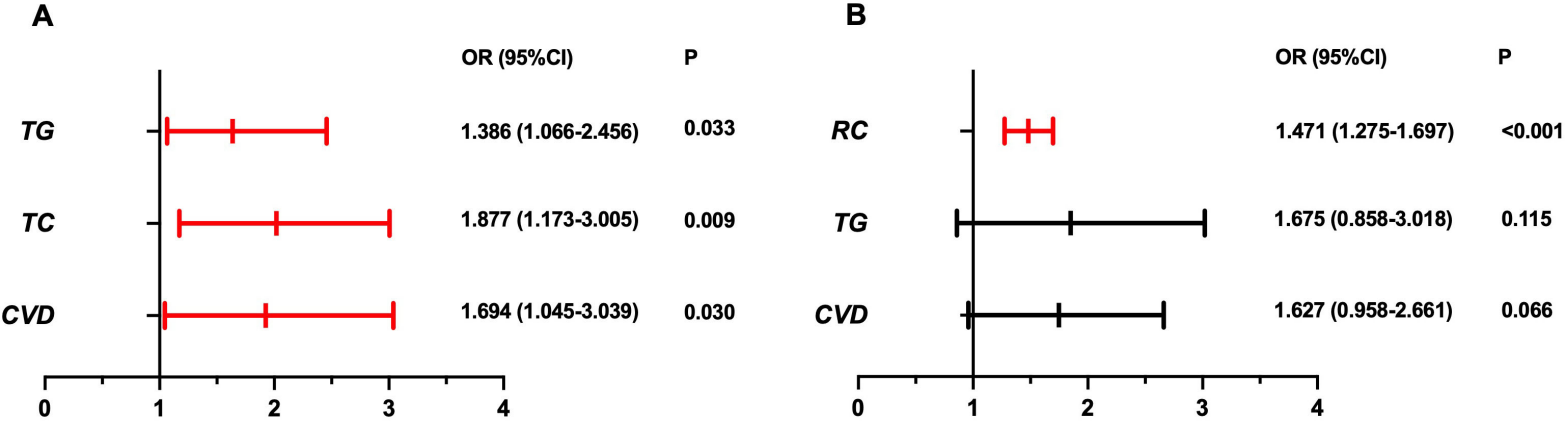
Multivariable logistic regression for AED. Forest plots illustrating odds ratios (ORs) with 95% confidence intervals (CIs) from logistic regression analyses. **(A)** Model 1. **(B)** Model 2.

The regression results are summarized in **Table 4**, and graphically displayed in **Figure 2**. In univariate logistic regression, TC, TG, CVD, and RC were significantly associated with AED. After adjusting for potential confounders, two multivariate models were constructed to address collinearity among lipid fractions. In Model 1 (including TC, but not RC), both TC (OR 1.877, 95% CI 1.173-3.005, P = 0.009) and TG (OR 1.386, 95% CI 1.066-2.456, P = 0.033) remained significant predictors of AED, along with CVD (OR 1.694, 95% CI 1.045-3.039, P = 0.030) (**Figure 2A**). In Model 2 (replacing TC with RC), RC showed a strong and independent association with AED (OR 1.471, 95% CI 1.275-1.697, P < 0.001), while TG and CVD were no longer significant (**Figure 2B**). These findings suggest that RC provides more robust information than traditional lipid fractions in predicting the presence of AED.

**Table 4:** Multivariate logistic regression analysis for AED compared with controls.

Variable	Univariate logistic regression		Model 1		Model 2	
	OR (95%CI)	P	OR (95%CI)	P	OR (95%CI)	P
Age	1.010 (0.972-1.050)	0.613	**/**	**/**	**/**	**/**
BMI	1.058 (0.931-1.202)	0.388	**/**	**/**	**/**	**/**
Smoking	1.173 (0.681-2.021)	0.565	**/**	**/**	**/**	**/**
CVD	1.540 (1.036-2.683)	**0.036**	1.694 (1.045-3.039)	0.030	1.627 (0.958-2.661)	0.066
TC	1.996 (1.265-3.150)	**0.003**	1.877 (1.173-3.005)	0.009	**/**	**/**
FBG	1.567 (0.949-2.587)	0.089	**/**	**/**	**/**	**/**
HDL-C	0.838 (0.289-2.429)	0.744	**/**	**/**	**/**	**/**
LDL-C	1.235 (0.715-2.135)	0.449	**/**	**/**	**/**	**/**
TG	1.740 (1.035-3.421)	**0.039**	1.386 (1.066-2.456)	0.033	1.675 (0.858-3.018)	0.115
TT	0.952 (0.893-1.016)	0.136	**/**	**/**	**/**	**/**
RC	1.456 (1.269-1.672)	**<0.001**	**/**	**/**	1.471 (1.275-1.697)	<0.001

Although TC, HDL-C, LDL-C, and RC were included in the univariate analyses, collinearity was observed (VIF > 2). Therefore, two models were constructed: Model 1 (age, BMI, smoking, CVD, FBG, TC, TG, TT) and Model 2 (age, BMI, smoking, CVD, FBG, TG, TT, RC), ensuring no multicollinearity (all VIF < 2).

AED, arteriogenic erectile dysfunction; ED, erectile dysfunction; BMI, Body Mass Index; CVD, cardiovascular disease; TC, Total Cholesterol; HDL-C, high-density lipoprotein cholesterol; LDL-C, Low-density lipoprotein cholesterol; TG, Triglyceride; TT, Total Testosterone; RC, Residual cholesterol. Bold indicates P < 0.05.

### ROC analysis of lipid parameters for AED

**Figure 3:**
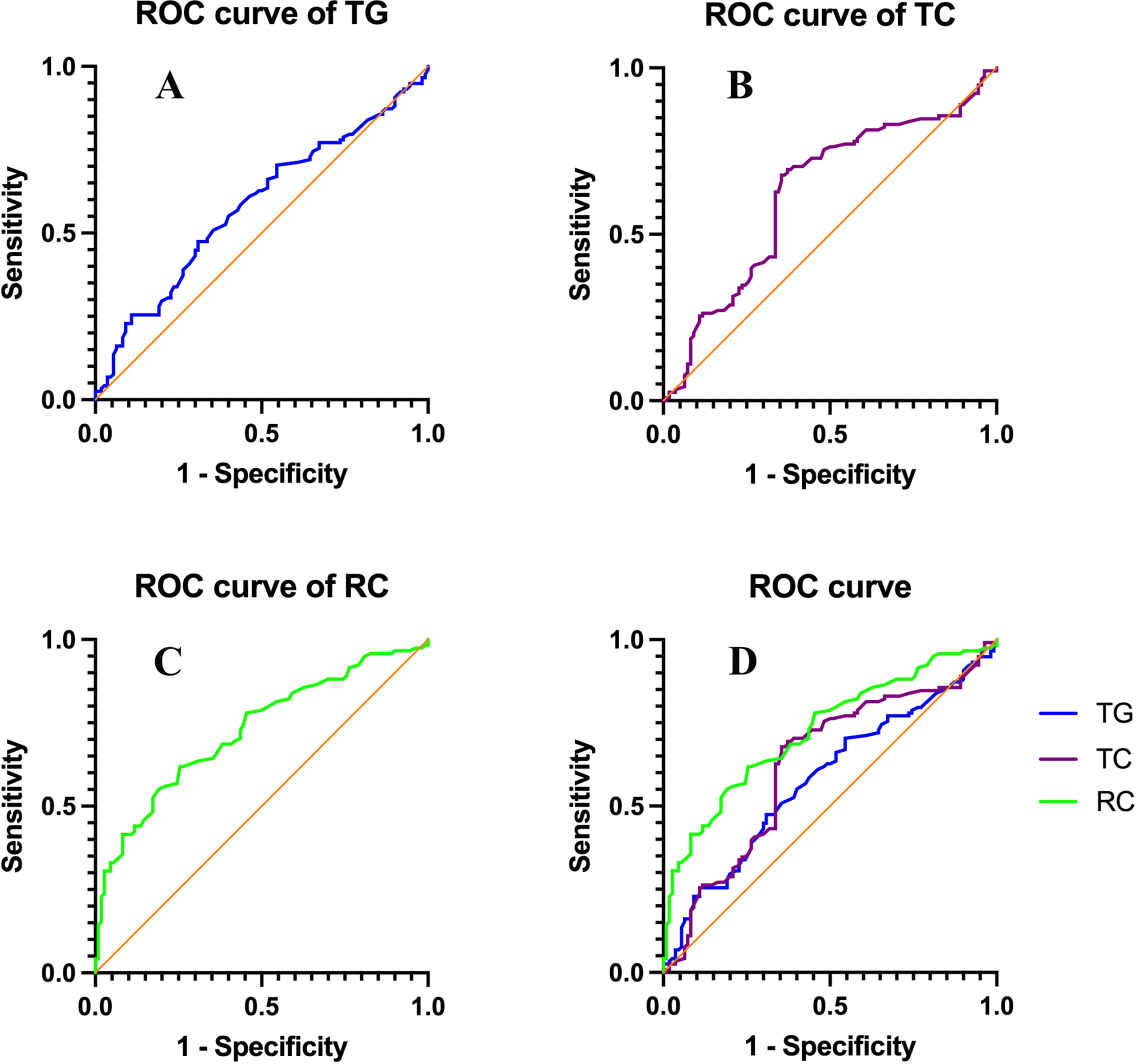
ROC curves of lipid parameters for predicting AED. Receiver operating characteristic (ROC) curves showing the predictive performance of lipid parameters in discriminating arteriogenic erectile dysfunction (AED) from controls. **(A)** ROC curve of TG; **(B)** ROC curve of TC; **(C)** ROC curve of RC; **(D)** Comparison of ROC curves for TG, TC, and RC.

ROC curve analyses were performed to evaluate the predictive value of TG, TC, and RC for arteriogenic ED. As shown in **Table 5**, RC demonstrated the best predictive performance with an AUC of 0.726 (95% CI 0.661–0.791, P <0.001), outperforming TC (AUC = 0.625, 95% CI 0.551–0.699, P = 0.001) and TG (AUC = 0.581, 95% CI 0.507–0.656, P = 0.034). The optimal cutoff value of RC was 0.595 mmol/L, yielding a sensitivity of 61.9% and a specificity of 74.5%. The ROC curves of the three lipid parameters are illustrated in **Figure 3**.

**Table 5:** Diagnostic performance of TG, TC, and RC for identifying AED.

Indicator	Optimal Cut-off (mmol/L)	Specificity	Sensitivity	AUC (95%CI)	P value
TG, mmol/L	1.425	69.1%	47.5%	0.581 (0.507-0.656)	0.034
TC, mmol/L	3.745	61.8%	69.5%	0.625 (0.551-0.699)	0.001
RC, mmol/L	0.595	74.5%	61.9%	0.726 (0.661-0.791)	<0.001

TG, Triglyceride; TC, Total Cholesterol; RC, Residual cholesterol; AED, arteriogenic erectile dysfunction; AUC, Area Under the Curve; CI, Confidence Interval.

## Discussion

In this study, we evaluated the associations of different lipid fractions with ED, with a particular focus on AED. Both TC and RC were elevated in patients with AED compared with controls. While TC showed some association with AED, RC demonstrated a stronger and more consistent relationship across multivariable models. Furthermore, ROC curve analysis indicated that RC had greater predictive value for AED than either TC or TG, with a higher AUC. In the overall ED population, RC levels were also higher than in controls, but the association was attenuated after adjustment for confounders. Taken together, these findings suggest that disturbances in cholesterol metabolism may be linked to ED in general, and that RC may represent a more specific marker of vascular impairment in men with AED.

Several studies have demonstrated a relationship between hyperlipidemia/dyslipidemia and ED (19). Epidemiological evidence has suggested that serum markers of lipid abnormalities, such as TG, TC, HDL-C, the HDL-C/TC ratio, and HDL-C and LDL-C levels, may serve as predictors of ED (2, 21–23, 36). Specifically, one clinical study on AED reported that elevated TG was associated with both AED and cardiovascular (CV) risk stratification (37). In contrast, research directly addressing RC in relation to ED remains limited. To date, only a single recent study has explored this topic, showing that higher RC levels were correlated with an increased prevalence of ED among men with diabetes, based on data from the NHANES database (30). While NHANES provides valuable population-based information, its limitations should be acknowledged, including reliance on a single self-reported question for ED diagnosis and the lack of information on medications that may affect erectile function or lipid metabolism. Compared with these findings, our results provide a meaningful extension by further examining the association between RC and AED in a clinically characterized cohort, offering more specific insights into the vascular subtype of ED.

Although direct mechanistic evidence linking RC to AED is lacking, several lines of data make the association biologically plausible. Elevated RC has been related to CVD independent of traditional risk factors (38), and ED is widely viewed as an early vascular warning sign (39); together they likely reflect shared pathophysiology (40). The artery-size hypothesis posits that atherosclerosis affects all vascular beds similarly, but smaller arteries occlude earlier; because penile arteries are narrower than coronary arteries, comparable degrees of endothelial dysfunction and plaque burden may translate into earlier flow limitation in the penis (41, 42). Beyond TC (43), remnant lipoproteins that constitute RC can traverse and be retained in the arterial intima, promoting macrophage lipid uptake, low-grade inflammation, and endothelial activation—processes central to impaired penile arterial inflow (44). RC elevation may also accompany triglyceride enrichment of LDL and the formation of small, dense LDL, further increasing atherogenicity (45). Consistent with this vascular profile, higher RC has been linked to greater peripheral arterial disease burden (46). Elevated RC has also been associated with insulin resistance and vascular inflammation, suggesting that metabolic dysregulation may amplify its vascular toxicity (47). Insulin resistance and impaired glucose handling can further aggravate endothelial injury by reducing nitric oxide bioavailability and increasing oxidative stress. Together, these findings imply that RC may act as a metabolic and inflammatory mediator linking dyslipidemia to vascular endothelial impairment. This convergence of lipoprotein remnant accumulation and metabolic disturbance provides a plausible explanation for the observed association between RC and AED, supporting the notion that AED could represent an early manifestation of systemic atherogenic processes. In addition, the role of RC may be viewed as a bridge between lipid metabolism and nitric oxide (NO)–mediated endothelial signaling. Lipoprotein remnants carried by RC can induce oxidative stress and promote endothelial nitric oxide synthase (eNOS) uncoupling, leading to reduce NO bioavailability and impaired endothelium-dependent vasodilation (48). The resulting imbalance between NO and reactive oxygen species (ROS) generation disrupts vascular homeostasis, favoring a pro-constrictive and pro-inflammatory state (49). Within the penile microcirculation, where erectile function relies heavily on rapid and sustained endothelium-dependent relaxation, such disturbances can markedly compromise arterial inflow and cavernosal smooth muscle relaxation (50, 51). Therefore, RC-driven endothelial dysfunction may directly contribute to penile vascular insufficiency, providing a potential mechanistic link between systemic atherogenic processes and localized AED. Taken together, these findings suggest a potential role of RC in the pathophysiology of AED.

In our regression analyses, an interesting difference was observed between the two multivariable models. In Model 1, which included TC but not RC, TG and CVD emerged as significant predictors of AED. However, in Model 2, where TC was replaced by RC, the associations of TG and CVD with AED were no longer statistically significant. This pattern suggests that the predictive information carried by TG and CVD may, at least in part, be captured by RC, underscoring the stronger relevance of RC in characterizing the vascular component of ED. Notably, the association between RC and AED persisted after further adjustment for fasting blood glucose, suggesting that RC acts as an independent vascular risk marker rather than a secondary reflection of metabolic disturbance. Taken together, these observations support a close relationship between RC and the vascular component of ED, particularly in patients with AED. Because RC is simple to calculate and reflects residual cardiovascular and metabolic risk beyond traditional lipid parameters, it may provide additional value in identifying ED patients at higher vascular risk. Our results suggest that RC could serve as a useful biomarker to refine risk stratification in men with AED. Nevertheless, current evidence remains limited, and prospective longitudinal studies are needed to confirm whether targeting RC reduction can translate into improved erectile or vascular outcomes.

The independent association of RC with arteriogenic ED (AED) and its superior discriminative value compared with TC and TG (AUC = 0.726) suggest that RC may serve as a practical biomarker for vascular risk stratification in men with ED. Because RC reflects residual atherogenic lipoproteins even in individuals achieving LDL-C goals, it may help identify patients with persistent vascular vulnerability that is not captured by conventional lipid indices. Incorporating RC assessment into the routine evaluation of men with ED could therefore provide additional insight into subclinical atherogenic burden and guide the need for more comprehensive cardiometabolic screening. Beyond risk assessment, recent RNA-based lipid-lowering therapies targeting hepatic PCSK9 expression have been shown to modulate both lipid and inflammatory pathways (52). Conceptually, these advances parallel the potential benefits of RC modulation, as both approaches aim to restore endothelial homeostasis and mitigate systemic atherogenic processes. The use of calculated RC represents both a practical advantage and an inherent limitation of the present study. Calculated RC, derived from standard lipid profiles (TC − HDL-C − LDL-C), is inexpensive, readily obtainable, and easily integrated into routine clinical and epidemiological assessments. These characteristics make it highly suitable for large-scale population studies and real-world clinical applications. However, this approach also has limitations. Because calculated RC is an indirect estimation, its accuracy depends on the precision of measured lipid components and the estimation formula. Unlike directly measured RC, which quantifies triglyceride-rich lipoprotein particles, the calculated RC may not fully capture particle heterogeneity or reflect postprandial lipid dynamics. However, previous studies have shown that calculated RC strongly correlates with directly measured RC and exhibits comparable predictive value for cardiovascular and metabolic outcomes (53). In large cohort analyses, calculated RC demonstrated similar associations with atherosclerotic cardiovascular disease, insulin resistance, and systemic inflammation as directly quantified remnant lipoproteins (28, 54). These findings support the prognostic reliability of calculated RC and justify its use in clinical and epidemiological research when direct measurement is not feasible.

Taken together, these perspectives underscore the translational potential of RC not only as a biomarker for early vascular impairment but also as a future therapeutic target within the evolving framework of metabolic–vascular protection.

Several limitations of this study should be acknowledged. First, its hospital-based, cross-sectional design precludes causal inferences, and residual confounding cannot be entirely excluded despite multivariable adjustment. Second, the sample size was relatively modest, particularly for the AED subgroup, which may limit statistical power and the generalizability of our findings. Third, although we carefully excluded individuals with major comorbidities or medications known to affect sexual function and lipid metabolism, unmeasured factors such as diet, and genetic predisposition were not assessed. Fourth, RC was calculated indirectly from standard lipid measurements rather than quantified by direct assays, which may introduce measurement bias. Finally, erectile function was evaluated by questionnaire and physiological testing but not confirmed by long-term follow-up, and the study population was limited to three hospitals in China, potentially restricting external applicability.

## Conclusions

In this hospital-based study, RC levels were elevated in men with ED and showed a stronger association with the arteriogenic subtype (AED) than with overall ED. RC demonstrated greater predictive value for AED compared with conventional lipid parameters, suggesting its potential utility as a biomarker for vascular risk stratification in this population. These findings add to the growing body of evidence linking RC to vascular health, but should be interpreted cautiously. Further prospective studies are warranted to confirm these associations and to clarify the role of RC in the clinical evaluation of ED.

## Data Availability

The raw data supporting the conclusions of this article will be made available by the authors, without undue reservation.
